# Long-Term Protective Effects of Methamphetamine Preconditioning Against Single-Day Methamphetamine Toxic Challenges

**DOI:** 10.2174/157015911795017344

**Published:** 2011-03

**Authors:** A.B Hodges, B Ladenheim, M.T McCoy, G Beauvais, N Cai, I.N Krasnova, J.L Cadet

**Affiliations:** 1 Molecular Neuropsychiatry Research Branch, Intramural Research Program, National Institute on Drug Abuse, NIH, DHHS, Baltimore, MD 21224, USA; 2 Morgan State University, Department of Psychology, Baltimore, MD 21251, USA

**Keywords:** Methamphetamine, striatum, dopamine, preconditioning.

## Abstract

Methamphetamine (METH) use is associated with neurotoxic effects which include decreased levels of dopamine (DA), serotonin (5-HT) and their metabolites in the brain. We have shown that escalating METH dosing can protect against METH induced neurotoxicity in rats sacrificed within 24 hours after a toxic METH challenge. The purpose of the current study was to investigate if the protective effects of METH persisted for a long period of time. We also tested if a second challenge with a toxic dose of METH would cause further damage to monoaminergic terminals. Saline-pretreated rats showed significant METH-induced decreases in striatal DA and 5-HT levels in rats sacrificed 2 weeks after the challenge. Rats that received two METH challenges showed no further decreases in striatal DA or 5-HT levels in comparison to the single METH challenge. In contrast, METH-pretreated rats showed significant protection against METH-induced striatal DA and 5-HT depletion. In addition, the METH challenge causes substantial decreases in cortical 5-HT levels which were not further potentiated by a second drug challenge. METH preconditioning provided almost complete protection against METH –induced 5-HT depletion. These results are consistent with the idea that METH pretreatment renders the brain refractory to METH-induced degeneration of brain monoaminergic systems.

## INTRODUCTION

Methamphetamine (METH) use is now a global epidemic because it has become easier to synthesize and administer [1, 2]. Chronic METH users suffer physiological harms, psychosis [3] and cognitive impairments [4]. METH also causes neurodegenerative changes in the brains of human addicts including decreases in the density of striatal dopamine transporter (DAT) [5, 6] and serotonin transporter (SERT) [7] observed in positron emission tomography (PET) studies. Post-mortem studies have also provided evidence of marked decreases in striatal dopamine (DA) and DAT levels in METH abusers [8]. 

METH-induced neurodegenerative effects have been studied extensively using animal models. These abnormalities include depletion of DA and its metabolites, 3,4-dihyroxyphenylacetic acid (DOPAC) and homovallic acid (HVA) levels, DAT density in the striatum and nucleus accumbens [9], decreases in the levels of serotonin (5-HT) and its metabolite, 5-hydroxyindoleacetic acid (5-HIAA), SERT density [10] as well as cell death *via* activation of apoptotic pathways [11-13]. 

The vast majority of animal models investigating the biochemical effects of METH have utilized regimens in which either moderate to large doses of METH were injected several times within short intervals on a single day or repeated exposure to high doses of METH over a period ranging from several days to several weeks [9, 14]. It has been suggested that these regimens might only model acute overdoses [9] because chronic METH users generally initiate drug use by taking small doses at variable intervals which they follow with gradual increases in the frequency and doses of METH intake [15, 16]. Animal models have indeed been developed in attempts to mimic those patterns of METH use and to evaluate the neurochemical effects of the drug on monoaminergic systems in the brain [17-21]. These models have shown that administration of repeated sub-toxic doses of METH can attenuate METH-induced damage in the striatum [17-19], nucleus accumbens [17], frontal cortex [17, 18, 21] and hippocampus [20,  21]. 

We have recently published data documenting the effects of METH preconditioning on monoamine levels measured at 24-hours after the toxic METH challenges [17, 19]. The present study reports on the effects of METH pretreatment in animals killed 2 weeks after the last METH injections. In addition, we report on the neurochemical effects of two separate drug challenges with toxic doses of METH.

## MATERIALS AND METHODS

### Animals

Male Sprague-Dawley rats (Charles River Laboratories, Raleigh NC) weighing approximately 350-400 g were habituated approximately one week prior to treatment. Animals were housed in polyethylene cages containing hardwood bedding in a humidity and temperature controlled room with a 12 hour light: dark cycle. Animals were given rat chow and water ad libitum. All animals use procedures were performed according to the National Institutes of Health Guide for the Care and Use of Laboratory Animals and were approved by the local Animal Care Committee.

### Drug Treatment and Tissue Collection

Following habituation, each rat was injected i.p with progressively higher doses of METH-hydrocholoride (NIDA, Baltimore, MD) or an equivalent volume of saline as described in Table **1**. The saline-pretreated group was further divided into three subgroups. The first subgroup received saline on both challenge days (SSS). The second subgroup was challenged with METH (5 mg/kg X 6, given 1 hour apart) as previously described (SSM) [19]. The third subgroup received two challenges of the same doses of METH with 2 intervening days (SMM) (see Table **1**). The animals pre-treated with METH received METH on both challenge days (MMM). This approach generated 4 groups. Animals were weighed three times per week during the preconditioning period to ensure accurate dosing. Animals were also weighed on both challenge days as well as one and five days following the last challenge dose. Tympanic temperatures were taken thirty minutes prior to the first injection and thirty minutes after every other injection on the second challenge day. The animals were sacrificed 14 days following the METH challenge by decapitation, their brains quickly removed and microdissected over ice, snap frozen on dry ice and stored at -80ºC until used in the HPLC analysis.

#### HPLC Analysis

To quantify monoamine levels in each brain region, HPLC with electrochemical detection was used. Striatal, cortical and hippocampal samples (N=8 per group) were ultrasonicated in 0.01M perchloric acid, then centrifuged at 20,000*g* for 15 min. Concentrations of norepinephrine (NE), DA, DOPAC, HVA, 5-HT, and 5-HIAA in brain tissue extracts of METH- and saline-treated rats were measured by HPLC with electrochemical detection as described earlier [22]. Concentrations of DA, DOPAC, HVA, 5-HT, and 5-HIAA were calculated and expressed as pg/mg of tissue weight and shown as percentages of control concentrations. Breeze (Waters Corp), a software program, was used to calculate peak height and to integrate known standards for the HPLC data.

### Statistical Analysis

Values, expressed as pg/mg wet tissue, for each monoamine or metabolite in each brain region were imported into StatView (SAS Institute) for statistical analysis. Statistical analysis was performed using analysis of variance (ANOVA) followed by Fisher’s protected least significant difference (PLSD) (StatView 4.02, SAS Institute, Cary, NC). Differences were considered significant at *p* < 0.05.

## RESULTS

### METH Caused Increases in Tympanic Temperature in the Rat

There were no significant differences in initial body temperature between any of the groups prior to the first injection. After the first injection, all groups challenged with METH had significantly higher body temperatures than the group treated with saline. These increases in body temperature persisted throughout the time of observation, as shown in Fig. (**1**).

### METH Preconditioning Protects Against METH-Induced Monoamine Depletion

#### Striatum

The effects of METH on DA and its metabolites, DOPAC and HVA, in the striatum are shown in Fig. (**2**). METH caused significant decreases in DA levels [F_(4,26)_ = 7.860, p = 0.003], see Fig. (**2A**). Post-hoc analyses revealed that the SSM group showed significant METH-induced decreases (-49.96%), with the second METH challenge not causing any further DA depletion (-55.62%) in the SMM group. The METH-pretreated rats were protected against METH challenge-induced DA depletion in comparison to the SSM and SMM groups, as shown in Fig. (**2A**). METH also caused decreases in DOPAC levels in the striatum [F_(4,26)_ = 5.360, p = 0.003], see Fig. (**2B**). The SSM group experienced significant METH-induced decreases compared to the METH-pretreated rats (-26.41%); the second METH challenge did not cause any further DOPAC depletion (-28.99%) in the SMM group. The METH–pretreated rats were protected against METH challenge-induced DOPAC depletion. HVA levels were not significantly affected after the drug challenge [F_(4,26)_ = 0.051, p = 0.995] (data not shown).

The effects of METH on 5-HT and its metabolite, 5-HIAA, are shown in Figs. (**2C** and **2D**). METH caused decreases in 5-HT levels in the striatum [F_(4,26)_ = 3.307, p = 0.035]. Post hoc analyses showed that the SSM group experienced significant METH-induced 5-HT decreases (-31.15%). A second METH challenge did not cause further 5-HT depletion (-35.23%) in the SMM group. The METH-pretreated rats were protected against METH challenge-induced 5-HT depletion in comparison to the SSM and SMM groups. 5-HIAA levels were significantly decreased (-38.47%) in the SMM group [F_(4,26)_ = 2.205, p = 0.013], see Fig. (**2D**).

#### Cortex

The effects of METH on the 5-HT system in the cortex are shown in Figs. (**3A** and **3B**). METH caused significant decreases in 5-HT levels in the cortex [F_(5,31)_ = 6.231, p = 0.0004]. The SSM group experienced significant METH-induced decreases (-46.03%), but a second METH challenge did not cause any further 5-HT depletion (-52.01%) in the SMM group. The METH-pretreated rats were protected against the METH challenge-induced 5-HT depletion. The effects of METH on the 5-HT metabolite, 5-HIAA, are shown in Fig. (**3B**). The METH challenge induced significant decreases in cortical 5-HIAA levels [F_(5,32)_ = 3.893, p = 0.007]. The SSM group showed significant METH- induced decreases (-27.69%), whereas a second METH challenge did not cause further 5-HIAA depletion in the cortex (-42.71%) (compare SSM to SMM group). The METH-treated rats were protected against METH challenge-induced 5-HIAA depletion (compare MMM to SSM and SMM groups).

#### Hippocampus

The effects of METH on hippocampal 5-HT and 5-HIAA are presented in Figs. (**3C** and **3D**). METH caused decreases in hippocampal 5-HT levels [F_(4,26)_ = 3.067, p = 0.034]. The SSM group showed significant METH-induced decreases (-25.06%), whereas a second METH challenge did not cause any further 5-HT depletion in the SMM group (-36.78%). The METH-treated rats were protected against METH challenge-induced 5-HT depletion in comparison to the SSM and SMM groups, as shown in Fig. (**3C**). The SMM group experienced significant METH-induced 5-HIAA decreases (-42.56%) compared to SSS, see Fig. (**3D**). The METH-treated rats were protected against METH challenge-induced 5-HIAA depletion in comparison to the SSM and SMM groups. 

## DISCUSSION

The main finding of the present paper is that METH preconditioning can protect against the long-term biochemical effects of a toxic dose of the drug. The protective effects of METH pretreatment were observed in animals killed 14 days after the METH challenge. METH-pretreated animals also had significantly higher body temperatures compared to controls. These data suggest that protection caused by METH-preconditioning does not depend on changes in temperature. These results are consistent with data from previous studies that have used different pretreatment paradigms [17-19, 23-25]. 

Neuroprotection reported in animals that underwent the process of METH preconditioning have included significant attenuation of METH-induced depletion of monoamines [17, 19, 24] as well as METH-induced abnormalities in the activities of tyrosine hydroxylase (TH) and trypotophan hydroxylase (TPH) [24]. METH pretreatment can also attenuate METH-induced striatal DAT [23, 25] and VMAT-2 reductions [25]. Thus, when taken together, these observations suggest that repeated injections of low doses of METH might render the brain refractory to the toxic effects of binge injections of the drug, thus providing protection against toxic METH effects in various brain regions.

We found, in addition, that a second challenge with a toxic METH regimen did not cause further reduction in monoamine levels. This suggests that the first challenge might have triggered protective mechanisms in the brain. These results are consistent with those of Hanson *et al.* [27] who recently reported that subsequent challenges with toxic doses of METH did not cause further abnormalities in striatal DA levels, DAT function, and VMAT-2 function. Mechanisms underlying this neuroprotection might include activation of antioxidant enzymes and chaperone proteins such as heat shock proteins [28]. The possibility that this pattern of METH might also interfere with the ability of mitochondria to generate toxic free radicals also needs to be considered [9]. More comprehensive studies are underway to test these ideas.

## Figures and Tables

**Fig. (1):**
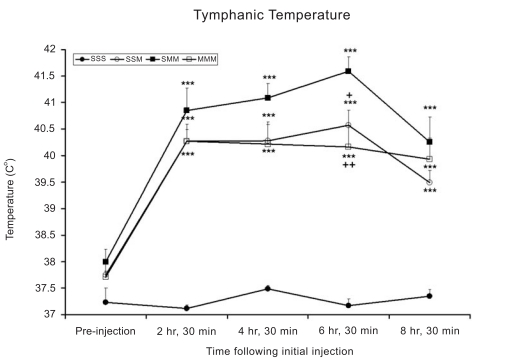
METH induced changes in tympanic temperatures in the rat. Temperatures were measured in all animals in the two treatment groups after injections of either METH (6 X 5 mg/kg, every hour) or saline challenges. Values are expressed as means ± SEM, N = 6-10 animals per group. Key to statistics: *** p < 0.001 versus SSS group; + p < 0.05 and ++ p < 0.01 versus SMM group.

**Fig. (2):**
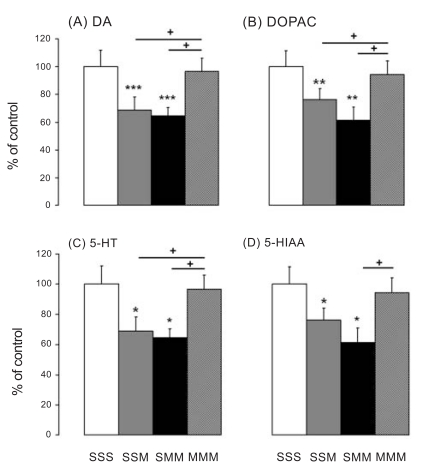
Effects of METH pretreatment and challenge on striatal DA (**A**), DOPAC (**B**), 5-HT (**C**), and 5-HIAA (**D**) levels. Values are expressed as percentages of the SSS group (mean ± SEM). N = 5-8 animals per group. Key to statistics: *p < 0.05, ** p < 0.01, and *** p < 0.0001 versus SSS group; +p < 0.05 versus MMM.

**Fig. (3):**
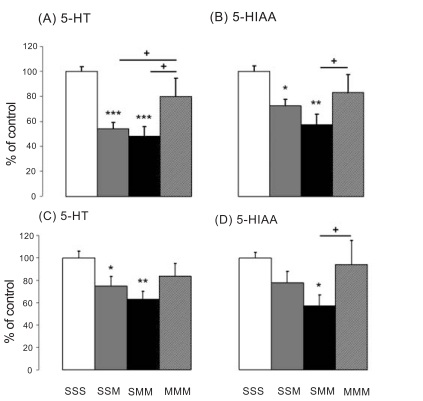
Effects of METH pretreatment and challenge on levels of cortical 5-HT (**A**) and 5HIAA (**B**) and of hippocampal 5-HT (**C**) and 5HIAA (**D**) after METH pretreatment and challenges. Values are expressed as percentages of the SSS group. Data are means ± SEM, n= 5-8 animals per group. Key to statistics: *p < 0.05 and ** p < 0.01 versus SSS group; +p < 0.05 versus MMM.

**Table 1:** Schedule of METH Pre-Treatment and Challenges

Week 1-Pretreatment							
	Monday	Tuesday	Wednesday	Thursday	Friday	Saturday	Sunday
9:00	0.5 mg/kg	1.0 mg/kg	1.0 mg/kg	1.5 mg/kg			
10:00							
11:00			1.0 mg/kg	1.5 mg/kg			
12:00							
13:00			1.0 mg/kg	1.5 mg/kg			
14:00							
15:00	0.5 mg/kg	1.0 mg/kg	1.0 mg/kg	1.5 mg/kg			
**Week 2 - pretreatment**							
9:00	1.0 mg/kg	1.5 mg/kg	2.0 mg/kg	2.5 mg/kg			
10:00							
11:00	1.0 mg/kg	1.5 mg/kg	2.0 mg/kg	2.5 mg/kg			
12:00							
13:00	1.0 mg/kg	1.5 mg/kg	2.0 mg/kg	2.5 mg/kg			
14:00							
15:00	1.0 mg/kg	1.5 mg/kg	2.0 mg/kg	2.5 mg/kg			
**Week 3-Challenge Doses**							
9:00	5.0 mg/kg			5.0 mg/kg			
10:00	5.0 mg/kg			5.0 mg/kg			
11:00	5.0 mg/kg			5.0 mg/kg			
12:00	5.0 mg/kg			5.0 mg/kg			
13:00	5.0 mg/kg			5.0 mg/kg			
14:00	5.0 mg/kg			5.0 mg/kg			

The rats were initially divided into two groups, with one group receiving saline and the other group getting METH pre-treatment according to the schedule described above during the first and second weeks.  Challenge doses were given either on Monday or Thursday of the third week or both days.  The saline pre-treatment was followed by either saline on both challenge days (SSS), METH on the second challenge day (SSM) or METH on both challenge days (SMM).  The METH pre-treatment was followed by METH challenges on both days (MMM).  All animals were killed two weeks later.
